# Neighborhood context shapes physical activity intervention outcomes: a comparison of human vs. virtual advisors

**DOI:** 10.1186/s12889-026-26885-5

**Published:** 2026-03-16

**Authors:** Astrid N. Zamora, Maria I. Campero, Dulce M. Garcia, Timothy Bickmore, Abby C. King

**Affiliations:** 1 Department of Epidemiology & Population Health, Stanford University School of Medicine, Stanford University, 1701 Page Mill Road, Palo Alto, Stanford, CA 94304 USA; 2 Khoury College of Computer Sciences, Northeastern University, Boston, MA USA; 3 Stanford Prevention Research Center, Department of Medicine, Stanford University School of Medicine, Stanford, CA USA

**Keywords:** Aging, MVPA, Older adults, Neighborhood, Physical activity, Walking

## Abstract

**Background:**

Neighborhood conditions are key social determinants of health (SDOH) that play a critical role in healthy aging. Incorporating contextual measures into behavioral medicine interventions is essential for creating adaptable, equity-focused health solutions. Yet, few physical activity (PA) interventions explicitly evaluate how these factors influence effectiveness. We conducted a secondary analysis of the Computerized Physical Activity Support for Seniors (COMPASS) Trial to examine whether neighborhood context, measured by the California Healthy Places Index (HPI), moderated intervention effects on PA among Latino/a older adults.

**Methods:**

The COMPASS trial was a single-blind, cluster-randomized non-inferiority trial comparing an interactive virtual advisor (reference arm) with a trained human peer advisor. PA outcomes, including weekly minutes of walking and moderate-to-vigorous physical activity (MVPA), were assessed with the CHAMPS questionnaire at baseline and 12 months. Neighborhood conditions were measured with the HPI, a composite index of 25 indicators across eight domains (e.g., housing, education, transportation) that reflect place-based SDOH. Scores range from 0 to 100, with higher values indicating more health-supportive environments. Mixed-effects ANCOVA models adjusted for baseline PA, age, and gender, with a random intercept for study site, and tested effect modification via interaction terms between intervention arm and HPI.

**Results:**

Among 245 Latino/a participants (mean age = 62.3 years; 78.8% female), HPI significantly moderated intervention effects. When modeled continuously, significant interactions were observed for 12-month changes in walking (β = − 2.93; *P* = .041) and MVPA (β = − 2.54 min/week per HPI point; *P* = .033). These findings indicated that the human advisor was comparatively more effective in lower-HPI neighborhoods, whereas the virtual advisor produced stronger improvements in higher-HPI neighborhoods. In sensitivity analyses using dichotomized HPI, the interaction was significant for MVPA (β = − 112.9; *P* = .026) and trended for walking (*P* = .077).

**Conclusions:**

Neighborhood context moderated the relative effectiveness of digital versus human-delivered PA interventions. These findings suggest that tailoring delivery strategies, leveraging digital tools in advantaged areas and peer support in under-resourced neighborhoods, may enhance equity in health promotion for older Latino/a adults by explicitly accounting for neighborhood-level social determinants of health.

**Trial registration:**

Clinicaltrials.gov NCT02111213 Registered April 2, 2014 https://clinicaltrials.gov/study/NCT02111213.

**Supplementary Information:**

The online version contains supplementary material available at 10.1186/s12889-026-26885-5.

## Introduction

In 2024, the Centers for Disease Control and Prevention reported that physical inactivity contributes to 1 in 10 premature deaths and is associated with $117 billion in annual health care costs in the United States (U.S.) [1]. However, the burden of physical inactivity is unevenly distributed, with the prevalence being higher among older adults, communities experiencing socioeconomic disadvantage [2], and racial and ethnic minorities (particularly Latino/a/Hispanic and Black adults in the U.S.) [3]. Older Latino/a adults are especially at risk of physical inactivity, as they are underrepresented in existing health promotion interventions and often lack access to culturally tailored and accessible physical activity programs [4]. These inequities contribute to disproportionately higher rates of obesity, type 2 diabetes, and cardiovascular disease [5, 6], chronic conditions that can be both prevented and managed through increased physical activity [7]. Beyond these health consequences, these disparities reinforce broader inequities in life expectancy, healthcare costs, and overall quality of life [8, 9].

Digital health interventions such as eHealth (e.g., computer-based programs) and mHealth (e.g., mobile phone-based tools) offer scalable and potentially cost-effective solutions for promoting physical activity [10, 11]. However, most digital health interventions have been designed for and tested among individuals who are younger, highly educated, and of non-Hispanic White descent [2]. As a result, these approaches may inadvertently exacerbate health disparities if they do not adequately engage populations at highest risk of physical inactivity and chronic disease.

Understanding the built environment and neighborhood context in which physical activity interventions are delivered is essential, as these conditions shape both intervention effectiveness and their potential to advance health equity. Physical activity is not solely an individual level behavior, it is shaped by broader social determinants of health (SDOH), including neighborhood safety, access to parks and recreation, walkability, and economic stability [12, 13]. To capture the multidimensional impacts of SDOH on community health, composite indices such as the California Healthy Places Index (HPI) have been developed [14, 15]. The California HPI was designed as a policy and planning tool to help stakeholders identify areas with the greatest need for investment in health-supportive infrastructure and services [14, 15]. It integrates data across multiple domains, including economic resources, housing, education, transportation, and healthcare access, to provide a summary measure of neighborhood conditions associated with life expectancy and health equity. Higher HPI scores indicate communities with greater resources and conditions that promote health and well-being.

Although some studies have examined associations between neighborhood environments and health outcomes, including physical activity, among Latino/a/Hispanic adults [16], evidence remains limited on how neighborhood-level conditions influence the effectiveness of physical activity interventions, particularly among older Latino/a adults in the U.S. This gap is especially relevant in California, which has both the largest number and one of the highest percentages of Latino/a residents in the U.S [17, 18]. Addressing neighborhood-level barriers, may be key to reducing health disparities and improving the reach and impact of physical activity programs.

The present study is a secondary analysis of a cluster-randomized non-inferiority trial that compared two culturally tailored physical activity interventions, delivered by either a computer-based virtual advisor or human peer advisors, over 12 months to aging Latino/a adults from the San Francisco Bay Area [19]. The primary trial found that the two interventions were similarly effective in increasing walking at 12 months [19]. Building on those findings, we examined whether neighborhood-level condition, measured by the California Healthy Places Index (HPI) [20], moderated intervention effectiveness. HPI captures multiple social and environmental factors relevant to physical activity, such as access to parks and transportation [21, 22]. We therefore hypothesized that intervention effects would differ by neighborhood context. In addition, we conducted exploratory analyses to assess within-arm associations between HPI and physical activity outcomes, peer advising session length by neighborhood context, and differences in technology comfort across the HPI group.

## Methods

### Study design and participants

This secondary analysis involved participants from the Computerized Physical Activity Support for Seniors (COMPASS) trial, a single-blind, cluster-randomized non-inferiority parallel trial conducted by the Stanford University School of Medicine and Northeastern University. The trial received Institutional Review Board approval at Stanford University, and all participants provided written consent in their preferred language (English or Spanish). The study adhered to the Consolidated Standards of Reporting Trials (CONSORT) guidelines, and a CONSORT flow diagram detailing participant flow was previously published [19].

The trial protocol is available at ClinicalTrials.gov (NCT02111213) and in the published protocol paper [23]. This secondary analysis was not prespecified in the original protocol.

Eligible participants were community-dwelling adults aged 50 years and older who were not meeting physical activity guidelines (< 100 min per week of moderate-intensity activity in the prior month, assessed using the Community Healthy Activities Model Program for Seniors [CHAMPS] instrument). They also needed to be able to safely engage in activities such as walking, reside within five miles of a study-designated community center, have sufficient English or Spanish literacy to provide informed consent and participate in study procedures (including computer use), and plan to remain in the area for at least one year [19].

Participants were recruited between July 2014 and July 2016 through geographically targeted mailings, culturally tailored media promotion, and community outreach in Santa Clara and San Mateo counties (California, U.S.). Community centers were matched by locale and randomized in pairs (1:1 allocation) to either the virtual advisor or human peer advisor intervention using a computerized randomization sequence. The final 12-month visit occurred in September 2017.

A total of 94.3% of participants (231 out of 245) completed the 12-month data collection for walking minutes per week (Virtual Advisor 95.1%; Peer Advisor 93.4%). Missing outcome data were addressed using imputation methods consistent with intention-to-treat principles. The complete methods have been previously described elsewhere [19].

### Intervention description

Both intervention arms were based on the evidence-supported Active Choices program, grounded primarily in Social Cognitive Theory and incorporating constructs from the Transtheoretical Model of behavior change. Core theoretical elements included enhancement of self-efficacy, use of self-regulatory strategies (e.g., goal setting and pedometer-based self-monitoring), problem-solving around barriers, reinforcement of progress, and mobilization of social support. Intervention content was tailored to participants’ readiness to change and individual goals across arms [24–28].

In both intervention arms, participants received up to 28 brief (approximately 10–15 min) one-on-one counseling sessions over 12 months, delivered weekly during the first two months and biweekly thereafter. Each session followed a structured protocol including review of recent walking behavior, discussion of barriers and facilitators, individualized goal setting, reinforcement of progress, and scheduling of the next session.

Participants in the virtual advisor arm interacted with the embodied agent via touchscreen prompts in English or Spanish, with real-time tailored feedback based on self-reported activity and pedometer uploads and simulated face-to-face counseling using synthetic speech and nonverbal cues [23, 29, 30].

In the peer advisor arm, trained community-based advisors delivered the same structured behavioral curriculum in person using standardized session guides. Peer advisors were community residents aged ≥ 30 years who engaged in regular moderate-intensity physical activity and completed a 12-hour standardized training program based on previously validated peer-led interventions [19, 25, 26]. Advisors participated in ongoing supervision meetings and quality assurance procedures throughout the study.

Both intervention arms incorporated culturally tailored messaging and bilingual delivery options.

### Physical activity outcome assessment

The primary outcomes were 12-month total walking minutes per week and MVPA minutes per week, assessed using the validated CHAMPS questionnaire [31, 32]. Walking change was derived from the four walking-specific CHAMPS items. CHAMPS was selected due to its demonstrated validity in older adults and its ability to capture specific physical activity types linked to public health guidelines. CHAMPS has been correlated with accelerometry and doubly labeled water–measured energy expenditure [33]. In the parent COMPASS trial, accelerometry was used to corroborate physical activity findings [19].

### California Healthy Places Index

The California Healthy Places Index (HPI) version 2.0 is a publicly available, composite measure of neighborhood-level conditions associated with health and life expectancy (https://www.healthyplacesindex.org*)* [15]. Developed by the Public Health Alliance of Southern California in collaboration with Virginia Commonwealth University, HPI version 2.0 integrates 25 standardized indicators across the following eight domains: education, housing, transportation, economic opportunity, pollution, built environment, healthcare access, and social environment. Each indicator is converted to a z-score, weighted, and aggregated to generate an overall index score. All indicators are scaled so that higher values reflect more health-supportive neighborhood conditions. HPI scores are reported as percentiles ranging from 0 to 100, with higher percentiles indicating healthier and more advantaged neighborhoods.

We selected HPI version 2.0 because its data collection period aligned with the COMPASS trial timeline. Participant residential ZIP codes were geocoded and matched to the corresponding HPI score. For the moderation analyses, we first modeled HPI as a mean-centered continuous variable to assess how intervention effects varied across the full range of neighborhood conditions. We then conducted a sensitivity analysis by dichotomizing HPI at a percentile cutoff of 50 (low vs. high) to assess the consistency of moderation patterns. We selected the 50th percentile as a pragmatic cutoff to distinguish between lower- and higher-resource neighborhoods in the absence of a validated threshold, and to ensure balanced sample sizes across groups. To further assess baseline comparability of neighborhood conditions by intervention arm, mean (± SD) values of three zip code level built environment indicators of particular relevance for PA were evaluated: (1) park access, defined as the percentage of residents living within a half-mile of a park, beach, or open space; (2) retail density, measured as the number of retail, entertainment, service, and education jobs per acre; and (3) tree canopy, calculated as the percentage of land covered by trees, weighted by population density. These subcomponents were presented alongside the overall HPI score to explore whether baseline environmental attributes relevant to walking and MVPA differed by intervention arm. A full description of HPI development and methodology has been published elsewhere [14, 34].

### Additional measures

At 12 months, participants completed two items from the validated Computer Attitude Scale [35] assessing perceived discomfort and difficulty with computers (5-point Likert scale; higher scores indicating more negative perceptions). Variables were retained in their original ordinal form for analysis.

### Statistical analysis

Baseline characteristics were summarized as mean ± SD (or median [IQR] for skewed variables) for continuous measures and as N (%) for categorical variables. Between-arm differences were evaluated using independent-samples t tests for continuous outcomes and Pearson’s χ² tests (or Fisher’s exact tests when expected cell counts were small) for categorical outcomes. Contextual measures (e.g., park access, retail density, tree canopy) were likewise compared using t-tests.

Neighborhood variability in HPI was illustrated with a ZIP code–level heatmap generated in R (version 4.3.1; R Core Team, 2024) using the ggplot2 and dplyr packages. Each participant’s residential ZIP code was matched to its corresponding HPI score (range 0–100), with darker colors representing less health-supportive neighborhoods and lighter colors representing more supportive neighborhoods. This visualization highlights the geographic diversity of neighborhood contexts represented in the sample and provides spatial context for the moderation analyses.

The primary moderation analysis used mixed-effects analysis of covariance (ANCOVA) models fit via PROC MIXED with restricted maximum likelihood estimation, with a random intercept to account for clustering by study site. Two physical activity outcomes were analyzed: (1) 12-month change in total walking minutes per week and (2) 12-month change in MVPA minutes per week, in separate models. Each model included the intervention arm (the virtual advisor [Carmen] as the reference vs. the human peer advisor), the corresponding baseline PA measure, and prespecified covariates: HPI score (mean-centered), age, and gender. Because HPI is a ZIP code–level composite measure, we did not adjust for ZIP code separately, as this would represent statistical overadjustment by conditioning on the same level of variance. Fixed-effect estimates are presented as adjusted mean differences with 95% confidence intervals (CIs) and two-sided P values. To evaluate whether neighborhood conditions modified intervention effects, we added an interaction term between intervention arm and mean-centered HPI score. A two-tailed α < 0.05 for the interaction was considered evidence of effect modification by neighborhood context.

We then repeated these models using a binary HPI variable defined by the sample median (HPI < 50 vs. HPI ≥ 50) to confirm the moderation findings under a low versus high HPI categorization. These models retained the same fixed effects and random intercept structure, with interaction tests and subgroup estimates obtained from the PROC MIXED solution output.

To further explore how neighborhood conditions influenced outcomes, we conducted within-arm analyses stratified by intervention arm (virtual advisor vs. human peer advisor). For each arm, we fit mixed-effects ANCOVA models assessing the effect of HPI (modeled as a mean-centered continuous variable) on 12-month MVPA and walking minutes per week, adjusting for baseline physical activity, age, gender, and clustering by study site. This approach allowed us to evaluate whether the relationship between neighborhood context and physical activity varied within each intervention arm, independent of the overall interaction effects. We report β coefficients with 95% CIs and P values for the HPI effect in each stratified model.

In supplementary analyses, we compared (1) mean peer-advising session length between participants from lower- versus higher-HPI neighborhoods and (2) technology comfort at 12 months between HPI groups. Technology comfort was assessed with two Likert-scale items (range, 1 [strongly disagree] to 5 [strongly agree]), with higher scores reflecting greater discomfort/difficulty. Mean values were compared between HPI groups using independent-samples t tests.

An α level of *P* < .05 (two-tailed) was considered statistically significant. All analyses were conducted in SAS 9.4 (SAS Institute, Cary, North Carolina).

## Results

### Baseline participant characteristics

Baseline characteristics were generally well balanced across intervention arms (Table 1), with no statistically significant differences in demographic, contextual, or clinical measures. The only exception was park access, which was higher in the virtual advisor arm than in the human advisor arm [median (IQR) = 95.3 (9.3) vs. 91.9 (10.8); *P* = .01]. Overall, participants (*N* = 245) were predominantly female (78.8%) and middle-aged (mean ± SD age, 62.3 ± 8.4 years). They had lived in the U.S. for a median of 50.0 years (IQR = 30.0), with 56.9% born in the U.S. and 48.0% married. Mean household size was 3.5 (SD = 2.2), and mean educational attainment was 12.8 ± 4.1 years; 18.4% reported an annual household income above $75,000. Mean BMI was 32.9 ± 8.3 kg/m², with 49.4% hypertensive (SBP 127.6 ± 18.0 mm Hg; DBP 73.1 ± 9.0 mm Hg). The mean Vitality Plus total well-being score was 34.6 ± 7.9 (range 10–50). Baseline physical activity levels were low: mean total walking for the sample was 70.0 ± 98.0 min/week (virtual 80.2 ± 109.3 vs. human 59.6 ± 84.4; *P* = .10), and mean MVPA was 31.8 ± 53.7 min/week (virtual 39.9 ± 60.7 vs. human 23.5 ± 44.2). The median HPI score was 69.4 (IQR = 36.4) and did not differ significantly between arms (*P* = .08), with 50.6% classified as high-HPI.

**Table 1:** Baseline characteristics of participants in the COMPASS trial

Characteristic	Full Sample (*N* = 245)	Virtual Advisor (*N* = 123)	Human Peer Advisor (*N* = 122)	*P* value
Demographics				
Age, years, mean (SD)	62.3 (8.4)	62.1 (8.3)	62.4 (8.5)	0.82
Sex, female, N (%)	193 (78.8)	98 (79.7)	95 (77.9)	0.72
Years in U.S., median (IQR)	50.0 (30.0)	50.0 (28.0)	47.0 (31.0)	0.79
US Born, N (%)	139 (56.9)	67 (54.2)	73 (59.7)	0.39
Marital status, married, N (%)	118 (48.0)	54 (43.9)	64 (52.1)	
Household size, mean (SD)	3.5 (2.2)	3.7 (2.5)	3.3 (1.8)	0.15
Education, years, mean (SD)	12.8 (4.1)	12.8 (4.0)	12.8 (4.3)	0.88
Income, N (%):				
<$ 5,000 - $34,999	32 (13.1)	17 (13.8)	15 (12.3)	0.97
$35,000 - $49,999	23 (9.4)	11 (8.9)	12 (9.8)	
$50,000 - $74,999	30 (12.4)	16 (13.0)	14 (11.5)	
> $75,000	45 (18.4)	21 (17.1)	24 (19.7)	
Refused, does not know, or missing	115 (46.9)	58 (47.2)	57 (46.7)	
Retired, %	113 (46.3)	51 (41.5)	62 (51.2)	0.12
Health and clinical markers				
BMI, mean (SD)	32.9 (8.3)	33.4 (8.8)	32.3 (7.9)	0.26
SBP, mean (SD)	127.6 (18.0)	126.4 (17.8)	128.8 (18.2)	0.79
DBP, mean (SD)	73.1 (9.0)	72.6 (9.7)	73.6 (8.3)	0.10
Hypertensive, N (%)	121 (49.4)	59 (48.0)	62 (50.8)	0.65
Vitality plus score, mean (SD)	34.6 (7.9)	34.5 (7.8)	34.8 (8.1)	0.77
PA and sedentary measures				
Total walking, min/week, mean (SD)	70.0 (98.0)	80.2 (109.3)	59.6 (84.4)	0.10
MVPA, min/week, mean (SD)	31.8 (53.7)	39.9 (60.7)	23.5 (44.2)	0.06
Sedentary time, min/week, mean (SD)	2769.9 (1446.2)	2867.3 (1410.1)	2671.7 (1480.9)	0.58
Watch TV, min/week, mean (SD)	821.7 (706.7)	897.0 (740.6)	745.8 (665.2)	0.23
California HPI measures				
Continuous score, median (IQR)	69.4 (36.4)	69.4 (29.7)	69.4 (45.3)	0.08
HPI Strata, N (%)				
* High (HPI score ≥ 50)*	159 (64.9)	81 (65.9)	78 (63.9)	0.75
* Low (HPI score < 50)*	86 (35.1)	42 (34.1)	44 (36.1)	
Park Access, median (IQR)	93.4 (10.3)	95.3 (9.3)	91.9 (10.8)	0.01
Tree Canopy, median (IQR)	6.0 (2.9)	5.6 (2.9)	6.8 (5.9)	0.81
Retail Density, median (IQR)	6.0 (5.3)	6.0 (3.4)	5.6 (5.7)	0.05

Values are mean (SD), median (IQR), or n (%). Percentages may not total 100 due to rounding

*Abbreviations: HPI* Healthy Places Index (high stratum defined as HPI score ≥ 50; low stratum as < 50), *BMI* Body mass index, *SBP* Systolic blood pressure, *DBP* Diastolic blood pressure, *MVPA* Moderate−to−vigorous physical activity, *SD* Standard deviation, *IQR* Interquartile range

### Geographic variation in neighborhood health contexts

**Figure1:**
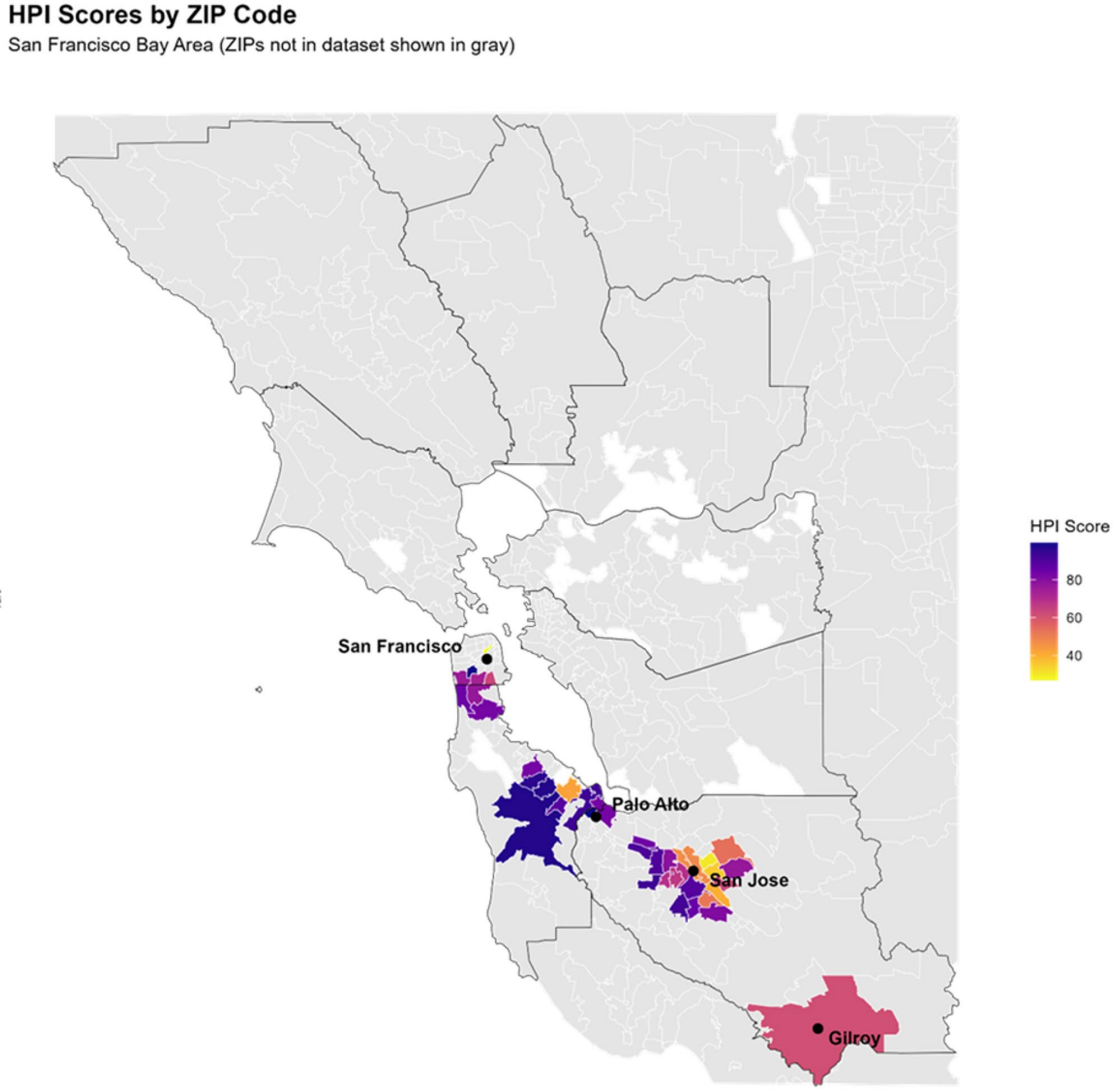
Geographic distribution of COMPASS trial participants' ZIP codes by California Healthy Places Index (HPI) score

Figure 1 presents a ZIP code–level heatmap of neighborhood HPI scores across the San Francisco Bay Area, created by matching each participant’s residential ZIP code to its HPI value. Scores ranged from 6.9 to 99.4 (median 69.4; SD 20.9; IQR 42.5–78.9). Cooler hues (purple–blue) correspond to higher HPI values and more health-supportive environments, whereas warmer hues (orange–yellow) correspond to lower HPI values and less supportive neighborhoods. Higher HPI areas clustered in affluent parts of northern Santa Clara County (e.g., Cupertino, Palo Alto) and San Mateo County. The city of San Jose exhibited substantial variability in HPI scores, with lower-HPI neighborhoods were concentrated in specific ZIP codes across the city (Fig. 1). This geographic heterogeneity reflects the diverse neighborhood contexts in which the interventions were implemented, and provides context for the moderation analyses.

### Primary moderation analysis with continuous HPI measure

In the primary analysis treating HPI as a continuous variable, neighborhood context moderated intervention effects for both walking and MVPA (Table 2). The intervention × HPI interaction was negative and significant for walking (β = − 2.93 min/week per one-point increase in HPI; *P* = .041) and for MVPA (β = − 2.54 min/week per one-point increase in HPI; *P* = .033). These effects indicate that the relative benefit of the virtual advisor increased in more advantaged neighborhoods, whereas the human advisor was comparatively more effective in lower-HPI settings. Independent of these interactions, higher HPI scores were also associated with greater average gains in walking (β = 2.17; *P* = .048) and MVPA (β = 2.01; *P* = .027) in models coded with Carmen as the reference arm, indicating that participants in more advantaged neighborhoods assigned to the virtual advisor experienced greater improvements.

**Table 2:** Fixed-effect model estimates for intervention, HPI, and intervention × HPI interactions on 12-month walking and MVPA

Physical Activity Outcome	Parameter	β (95% CI)	*P* value
MVPA (min/week)	Intervention (Human vs. Carmen at mean HPI)	-13.7 (-61.7, 34.3)	0.575
	HPI Score (main effect in Carmen arm)	2.01 (0.23, 3.79)	0.027
	Intervention × HPI Score	-2.54 (-4.88, -0.21)	0.033
Walking (min/week)	Intervention (Human vs. Carmen at mean HPI)	-39.6 (-99.8, 20.7)	0.197
	HPI Score (main effect in Carmen arm)	2.17 (0.02, 4.32)	0.048
	Intervention × HPI Score	-2.93 (-5.73, -0.13)	0.041

Intervention arm coded with Carmen (virtual advisor) as the reference; HPI score mean-centered. Models adjusted for baseline physical activity (same outcome), age, and gender, with a random intercept for study site. Estimates represent adjusted β coefficients (95% CI)

### Sensitivity analysis using dichotomized HPI measure

When HPI was dichotomized (lower < 50 vs. higher ≥50), the results were consistent with the primary analysis (Table 3). For MVPA, the intervention × HPI interaction was significant (β = − 112.9 min/week; *P* = .026), indicating that the virtual advisor produced greater improvements in higher-HPI neighborhoods. For walking, the interaction trended in the same direction but did not reach statistical significance (*P* = .077).

**Table 3:** Fixed-effect estimates from mixed-effects models examining intervention x HPI strata (lower vs. higher) interactions on 12-month walking and MVPA

Physical Activity Outcome	Parameter	β (95% CI)	*P* value
Walking (min/week)	Human vs. Carmen (lower-HPI)	32.2 (–65.3, 129.7)	0.516
	Higher- vs. lower-HPI (Carmen arm)	65.1 (–19.4, 149.7)	0.130
	Interaction: (Human vs. Carmen) × HPI stratum	–106.4 (–224.6, 11.8)	0.077
MVPA (min/week)	Human vs. Carmen (lower-HPI)	64.0 (–15.8, 143.7)	0.116
	Higher- vs. lower-HPI (Carmen arm)	80.8 (10.0, 151.5)	0.026
	Interaction: (Human vs. Carmen) × HPI stratum	–112.9 (–212.3, − 13.6)	0.026

Models adjusted for baseline physical activity (same outcome), age, and gender, with a random intercept for study site. HPI strata were defined by the sample median: low-HPI (<50) vs. high-HPI (≥50). Estimates represent adjusted mean differences (95% CI) in 12-month outcomes between the human peer advisor and the virtual advisor, stratified by neighborhood HPI level

### Exploratory within-arm associations

Exploratory models stratified by intervention arm did not show statistically significant associations between HPI and physical activity outcomes in either the human advisor or virtual advisor groups (Table 4).

**Table 4:** Within-arm associations between HPI scores and 12-month walking and MVPA

Intervention Arm	Physical Activity Outcome	β (95% CI )	*P* value
Human Peer Advisor	Walking (min/week)	2.03 (–0.49, 4.55)	0.113
	MVPA (min/week)	2.05 (–0.05, 4.14)	0.055
Virtual Advisor	Walking (min/week)	–0.96 (–2.33, 0.42)	0.171
	MVPA (min/week)	–0.20 (–1.53, 1.13)	0.763

Mixed−effects models fit separately within each intervention arm (human peer advisor vs. virtual advisor), adjusting for baseline physical activity (same outcome), age, gender, and clustering by study site. HPI scores range from 0–100, with higher scores indicating more advantaged neighborhoods. Positive β coefficients indicate greater physical activity with higher HPI scores, while negative coefficients indicate greater physical activity with lower HPI scores

### Exploratory analysis of peer session length

The mean length of peer advising sessions was slightly longer among participants from lower-HPI neighborhoods compared with those from higher-HPI neighborhoods, though this difference did not reach statistical significance (21.1 vs. 19.8 min; *P* = .056).

### Exploratory analysis of technology comfort

At the 12-month follow-up, participants from lower-HPI neighborhoods reported significantly greater discomfort using computers (mean score 2.71 vs. 2.31; *P* = .048) and greater perceived difficulty/frustration with computers (mean score 2.81 vs. 2.36; *P* = .025) compared with participants from higher-HPI neighborhoods. Scores were on a 1–5 Likert scale (1 = strongly disagree, 5 = strongly agree), with higher values indicating more negative perceptions of computer use. These results are reported narratively and are not shown in a table.

## Discussion

In this secondary analysis of older Latino/a adults enrolled in a 12-month cluster-randomized physical activity intervention trial, we found that neighborhood context, as measured by the California HPI, moderated the effects of the interventions on both walking and MVPA. The relative effectiveness of the human versus virtual advisor differed by neighborhood enviroment. Participants in lower-HPI neighborhoods assigned to the human peer advisor tended to show more favorable outcomes, although contrasts were not always statistically significant. In contrast, in more advantaged, higher-HPI neighborhoods, the virtual advisor produced significantly greater improvements in physical activity, particularly MVPA, suggesting that digital delivery was more effective in more resource-rich neighborhoods. When examined separately within each intervention arm, associations with HPI were null or inconsistent, reinforcing that the moderation effect was best captured by comparing the two interventions directly rather than by considering within-arm trends in isolation. Importantly, these patterns were not explained by individual-level income, which was balanced across study arms. Instead, HPI captured neighborhood-level differences in conditions such as infrastructure and amenities beyond what income alone could account for, underscoring its added value as a contextual moderator.

The present study highlights how neighborhood context may shape the effectiveness of behavior change interventions. In lower-HPI neighborhoods, where key social determinants such as access to safe walking environments, transportation, and economic stability may be more limited, human peer advisors may have been better positioned to provide flexible, context-sensitive support [19, 36, 37]. In contrast, Carmen, the virtual agent, delivered scripted messages via a touchscreen interface with pre-programmed nonverbal cues (e.g., facial expressions, gestures) [19, 23, 29], with limited capacity for adapting to specific structural constraints. The absence of such contextual tailoring may have reduced effectiveness in more disadvantaged settings, although this mechanism was not directly measured. Session lengths were on average longer among participants in lower-HPI neighborhoods, with a marginal trend (*P* = .056), which could reflect greater discussion of contextual barriers; however, this should be interpreted cautiously.

In higher-HPI neighborhoods, where environmental supports for physical activity (e.g., parks, walkable infrastructure) may be more readily available [38], digital delivery may have been sufficient to support behavior change without the need for more intensive human interaction. Scheduling flexibility with the virtual advisor, who was available throughout center hours, may also have contributed to its relative effectiveness in these settings, although this cannot be disentangled from broader neighborhood conditions. Exploratory analyses indicated that participants in lower-HPI neighborhoods reported greater computer-related discomfort, which may have influenced engagement with digital delivery. Taken together, these findings suggest that the relative effectiveness of human versus digital delivery may depend on neighborhood context rather than one modality being universally superior. Because HPI was applied retrospectively and potential mechanisms (e.g., resource navigation, digital comfort, session length) were not formally tested, these interpretations should be considered exploratory and hypothesis-generating.

Potential mechanisms underlying the observed moderation effects remain speculative. Two exploratory findings, a trend toward longer session length among lower-HPI participants and significantly greater computer-related discomfort in this group, suggest possible pathways. Although Carmen was designed to simulate a face-to-face interaction rather than a conventional computer interface, broader digital comfort may still influence how participants engage with technology-delivered interventions. These patterns were not tested as mediators and should be interpreted cautiously. Moreover, while HPI captures a broad set of neighborhood conditions across domains such as housing, transportation, and economic resources [15], it does not directly measure factors like perceived neighborhood safety, social cohesion, or the quality of walking environments. These unmeasured contextual influences may also have contributed to differences in intervention effectiveness. Together, these considerations highlight potential explanatory pathways while underscoring the need for future research to examine them more rigorously.

Our results echo prior findings comparing human versus automated advisors. For example, in a trial of older adults receiving physical activity advice via trained human counselors versus a human-sounding interactive voice response (IVR) system, participants with lower baseline motivation benefited more from human advisors, whereas those with higher baseline motivation did better with the automated system [39]. Although neighborhood context was not assessed in that study, the parallel is conceptually informative. Both studies suggest that the relative effectiveness of human versus automated delivery may depend on contextual or participant characteristics, rather than one modality being universally superior.

Taken together, these findings suggest that neighborhood context may shape the relative effectiveness of intervention modality. Importantly, the Healthy Places Index (HPI) was applied retrospectively in this secondary analysis and was not part of the original trial design. Therefore, these findings should be interpreted as exploratory and hypothesis-generating. Rather than suggesting that digital and human-delivered interventions are universally more or less effective in specific environments, our results indicate that intervention-context alignment warrants further prospective investigation. It is possible that in neighborhoods with greater structural resources (e.g., park access, walkability), scalable digital tools may be sufficient to support behavior change, whereas in lower-resource contexts, additional relational or structural support may enhance engagement. Future trials designed to stratify by neighborhood-level conditions are needed to test these hypotheses directly. Hybrid models integrating digital scalability with culturally grounded human support may represent a promising direction for equitable intervention design.

### Strengths and limitations

This study has several strengths. It focuses on older Latino/a adults, a population underrepresented in physical activity intervention research, thereby extending prior work in other groups. Use of the California Healthy Places Index provided a validated, area-based measure of neighborhood context that captures structural and environmental factors beyond individual socioeconomic status [34]. The cluster-randomized design and 12-month follow-up enhanced rigor, and the comparison of a culturally grounded human advisor with a scalable digital advisor provided insight into equity considerations in technology-supported health promotion.

Several limitations should be noted. This was a secondary analysis, and moderation by the California HPI was not a prespecified outcome, so findings should be viewed as hypothesis-generating. Physical activity was assessed by self-report, although we used the validated CHAMPS questionnaire, which correlates with objective measures, including accelerometry and doubly labeled water [33]. Session length and technology comfort were explored as potential explanatory factors, but were not directly tested, and observed differences were modest. The study sample was limited to older Latino/a adults in the San Francisco Bay Area, potentially reducing generalizability of our findings to older Latino/a adults in other parts of the country or world. Finally, while the California HPI is a valid measure, it does not capture all aspects of neighborhood context, and unmeasured factors may have influenced the present study results.

## Conclusions

Despite these limitations, the present findings offer novel exploratory evidence that neighborhood context may shape the relative effectiveness of human versus virtual-advisor delivered behavioral interventions. In conclusion, this study suggests that neighborhood environments may be associated with differences in outcomes between digitally versus human-delivered physical activity interventions among older Latino/a adults. Tailoring intervention modality to local conditions, such as neighborhood walkability, access to green space, and comfort with technology, may warrant consideration to advance equity in physical activity promotion. Future research should examine these potential differences in larger, more diverse samples and explore mechanisms linking neighborhood environments to intervention outcomes, while also considering scalable, sustainable implementation strategies.

## Supplementary Information

Supplementary Material 1.

## Data Availability

The dataset analyzed during the current study is not publicly available. However, upon reasonable request, the corresponding author (astridz@stanford.edu) can provide data access.

## Abbreviations

COMPASS: Computerized Physical Activity Support for Seniors
HPI: Healthy Places Index
PA: Physical Activity
